# Practical Notes on Diagnosis and Treatment

**Published:** 1908-01-11

**Authors:** 


### Practical Notes on Diagnosis and Treatment.

**Asthma in Children.**

To prevent nocturnal paroxysms give antipyrin, phenacetin, or bromides at bedtime, with small doses of heroin during the day.—*Dr. Cautley.*

**Syphilis of the Nervous System.**

Serious nerve lesions, producing, for example, paraplegia or hemiplegia, may appear at an early stage of the syphilitic infection. Here vigorous treatment is indicated, and the intra-muscular method is of distinct value.

**Epilepsy.**

Calcium carbonate is said to be of value in the treatment of epilepsy; a cachet containing 10 grains may be given twice daily. The fits are diminished and the patients are less excitable during the administration of this drug.

**Antipyrin in Nocturnal Incontinence.**

Dr. Leslie Phillips gives a single dose of antipyrin at bedtime in cases of nocturnal incontinence. Eight to ten grains may be ordered for a child of seven years. The drug may be used for months with good effect. As improvement sets in arsenic should be prescribed.

**Suppression of Urine.**

For the suppression of urine which sometimes follows surgical operations Dr. Stuart McGuire advises sulphate of sparteine. A dose of one to two grains is given hypodermically, and is repeated every three to six hours. The drug increases the blood-pressure and acts as a powerful diuretic.

**Amyl Nitrite in Hæmoptysis.**

If called to a case of hæmoptysis make the patient inhale the contents of a 3-minim capsule of amyl nitrite, and the bleeding usually stops at once, though clotted blood which has already been effused may continue to be coughed up. Afterwards, if the patient seems excited or alarmed, give a hypodermic injection of morphine.

**The Umbilical Cord.**

To prevent infection and to secure rapid separation of the umbilical cord it should be treated antiseptically. It should be tied with a sterile ligature, the scissors used to divide it should be sterile, and the dressing should consist of sterilised gauze.

**Unna's Zinc Jelly.**

Zinc oxide and gelatine of each 30 parts, glycerine and distilled water of each 50 parts. The jelly is melted in a water-bath, and then while fluid is painted on with a brush, some simple dusting powder is sprinkled over the surface as it dries.

**Obstinate Hiccough.**

The following methods have been found useful in individual cases:—1. A tablet of nitro-glycerine, $\frac{1}{100}$ grain, repeated every three or four hours. 2. A full dose of oil of turpentine made into an emulsion with mucilage has been followed by immediate relief. 3. Blistering each side of the neck over the origin of the third, fourth, and fifth cervical nerves.

**Uterine Inertia.**

In lingering labour due to uterine inertia quinine may be employed to increase the force and frequency of the uterine contractions Five to 10 grains may be given at intervals of half an hour for three doses. The drug may be ordered in cachets or suspended in milk; in the latter form it may be administered as an enema.

**Mongolian Blue Patches.**

These occur on the skin in about 90 per cent. of Japanese children. They are dark blue in colour, not raised above the surface, have an indefinite margin, and do not disappear on pressure. They are more marked in males than in females. Occasionally a child of European parents presents such patches, and the suggestion is that this means a previous crossing with some Mongolian strain.—*Dr. Ernest Jones.*

